# Crystal Structure of *Escherichia coli* Agmatinase: Catalytic Mechanism and Residues Relevant for Substrate Specificity

**DOI:** 10.3390/ijms22094769

**Published:** 2021-04-30

**Authors:** Pablo Maturana, María S. Orellana, Sixto M. Herrera, Ignacio Martínez, Maximiliano Figueroa, José Martínez-Oyanedel, Victor Castro-Fernandez, Elena Uribe

**Affiliations:** 1 Departamento de Biología, Facultad de Ciencias, Universidad de Chile, Ñuñoa 7800003, Santiago, Chile; pmaturana@ug.uchile.cl (P.M.); sixto.morales@ug.uchile.cl (S.M.H.); 2 Facultad de Ciencias de la Vida, Universidad Andres Bello, Santiago 8370251, Santiago, Chile; morellana@unab.cl; 3 Departamento de Bioquímica y Biología Molecular, Facultad de Ciencias Biológicas, Universidad de Concepción, Casilla 160-C, Concepción 4070386, Concepción, Chile; ignmartinez@udec.cl (I.M.); maxifigueroa@udec.cl (M.F.); jmartine@udec.cl (J.M.-O.)

**Keywords:** agmatine, agmatinase, ureohydrolase, enzyme specificity, arginase

## Abstract

Agmatine is the product of the decarboxylation of L-arginine by the enzyme arginine decarboxylase. This amine has been attributed to neurotransmitter functions, anticonvulsant, anti-neurotoxic, and antidepressant in mammals and is a potential therapeutic agent for diseases such as Alzheimer’s, Parkinson’s, and cancer. Agmatinase enzyme hydrolyze agmatine into urea and putrescine, which belong to one of the pathways producing polyamines, essential for cell proliferation. Agmatinase from *Escherichia coli* (EcAGM) has been widely studied and kinetically characterized, described as highly specific for agmatine. In this study, we analyze the amino acids involved in the high specificity of EcAGM, performing a series of mutations in two loops critical to the active-site entrance. Two structures in different space groups were solved by X-ray crystallography, one at low resolution (3.2 Å), including a guanidine group; and other at high resolution (1.8 Å) which presents urea and agmatine in the active site. These structures made it possible to understand the interface interactions between subunits that allow the hexameric state and postulate a catalytic mechanism according to the Mn^2+^ and urea/guanidine binding site. Molecular dynamics simulations evaluated the conformational dynamics of EcAGM and residues participating in non-binding interactions. Simulations showed the high dynamics of loops of the active site entrance and evidenced the relevance of Trp68, located in the adjacent subunit, to stabilize the amino group of agmatine by cation-pi interaction. These results allow to have a structural view of the best-kinetic characterized agmatinase in literature up to now.

## 1. Introduction

Agmatine is a primary amine product of decarboxylation of L-arginine, a reaction catalyzed by the enzyme arginine decarboxylase and initially described in bacteria and plants as a relevant precursor for the synthesis of polyamines, essential molecules for cell growth and development [1]. In 1994, Li et al., found agmatine in bovine brains as an endogenous agonist for imidazoline receptor and alpha-2 adrenergic receptor [2]. The evidence that this molecule plays essential roles at physiological and metabolic levels in mammals has been increasing, such as an inhibitor of nitric oxide synthase [3,4,5,6,7], a neurotransmitter in nicotinic receptor [8], a modulator of insulin release [9,10,11], and in renal excretion of sodium [12,13]. A neuroprotective effect and increased tolerance to morphine have been found with agmatine [14,15,16,17]. However, information on substrate specificity, structure, and evolution of the enzymes involved in the synthesis and degradation of agmatine is scarce.

Agmatinase (AGM) is the enzyme that catalyzes the hydrolysis of agmatine in urea and putrescine, and to date, *E. coli* AGM (EcAGM) is the best agmatinase kinetically characterized [18,19,20]. The K_M_ for its substrate agmatine is 1.1 mM [19], the *K_i_* for its competitive inhibitors arginine, guanidine, and putrescine are 9 mM, 15 mM, and 2.8 mM, respectively, and the catalytic constant (*k_cat_*) is 140 s^−1^ [19]. Crystal structures for the AGM from *Deinococcus radiodurans* [21] (PDB entry 3LHL) and the two putative AGMs from *Clostridium difficile* (PDB entry 3LHL) and *Burkholderia thailandensis* (PDB entry 4DZ4) [22] have provided insight into the active site that can accommodate two Mn^2+^ ions. Six residues that form the Mn^2+^ binding site are highly conserved among AGMs, including the human enzyme [20,23]. Regarding EcAGM, by mean of site-directed mutagenesis His126 and His151, were characterized as ligands of the activating metal Mn^2+^. His151 coordinates a strongly bound Mn^2+^ in the active site and His126 coordinates a weakly bound Mn^2+^ [24], which strongly support the hypothesis that the minimum requirement for AGM activity is one Mn^2+^ ion per active site, with a metal ion residing in a high-affinity site. Upon mutation of His163 by phenylalanine, the *E. coli* AGM activity was reduced to 3–5% of wild-type activity, without any change in K_M_ for agmatine or Ki for putrescine inhibition [25], which support that His163 would play an essential role in the catalytic function of AGM, proposing that it would participate in the stabilization of the transition state and in a proton shuttle to the putrescine product [25]. Upon mutation of Asp153 by asparagine, the catalytic activity of *E. coli* AGM was reduced to about 5% of wild-type activity. Asp153 is proposed to be involved, by hydrogen bond formation, stabilizing and orienting a metal-bound hydroxide for an optimal attack on the guanidinium carbon of agmatine. Thus, the disruption of this hydrogen bond is the likely cause of the significantly decreased catalytic efficiency of the D153N mutant [26].

AGMs belong to the ureohydrolases enzymes family, containing all the conserved residues of this family similar to arginases (ARG), proclavaminic acid amidino hydrolases (PAH), guanidinopropionase (GpuA), and guanidinobutyrase (GbuA) [21,27,28]. Arginase and agmatinase catalyze a similar reaction of hydrolysis of a guanidine group, producing urea and ornithine in the case of arginases. Arginases have been extensively studied in various species, being human ARG one of the best-characterized enzymes, kinetically and structurally, in this family. In the Protein Data Bank (PDB) database, there are more than 70 structures of ARG of more than ten different species. Only four x-ray structures have been determined for putative AGMs; *Burkholderia thailandensis* (PDB 4DZ4), *Clostridium difficile* (PDB 3LHL), *Thermoplasma volcanium* (PDB 3PZL), and *Deinococcus radiodurans* (PDB 1WOG), of which the unique AGM reported with agmatinase activity is DrAGM. However, the study does not report any kinetics parameters or substrate specificity characterization [21].

Unfortunately, reports of AGMs with crystal structures often lack detailed kinetic studies, the analysis of the residues that participate in the catalysis, and the possible determinants of agmatine specificity. Regarding substrate specificity, human ARG type I (hARG) is highly specific for arginine. However, the mutation of Asn130 to aspartate generated a mutant active with arginine and agmatine. Moreover, it exhibited no preferential substrate specificity for arginine over agmatine *k_cat_* ⁄K_M_ values of 2.48 × 10^3^ M^−1^ s^−1^ and 2.14 × 10^3^ M^−1^ s^−1^, respectively [29]. These results strongly suggest this asparagine residue as a determinant of specificity for arginases. Here, to analyze the residues involved in the specificity of EcAGM, different mutants of two loops at active site entrance were generated and characterized. Also, the crystal structures of *Ec*AGM were determined, one complexed with guanidinium and the other with urea and agmatine at the active site, which allowed us to analyze the reaction mechanism and oligomerization interfaces of *Ec*AGM. By molecular docking of agmatine in the active site and molecular dynamics simulations, the conformational dynamics of *Ec*AGM and residues participating in non-binding interactions were evaluated.

## 2. Results and Discussion

### 2.1. Mutagenesis Studies in E. coli Agmatinase

**Figure 1:**
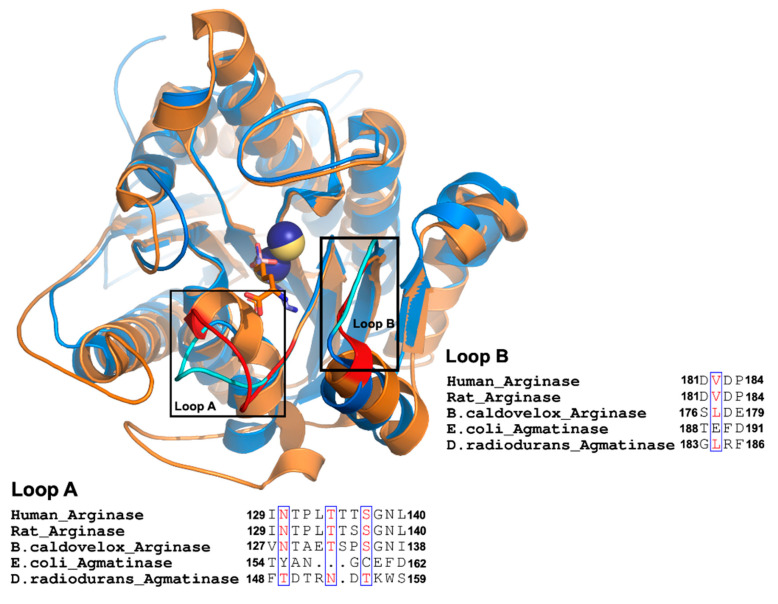
Comparison between agmatinases and arginases. Structural superposition of DrAGM (1WOG) and hARG-I (2AEB). DrAGM and hARG-I are represented in blue and orange, respectively. Loop A and B from DrAGM are shown in cyan, Mn2+ in dark blue, and hexane-1,6-diamino ligand is shown as sticks with blue carbons. Loop A and B from hARG-I are shown in red, Mn2+ ions in yellow, and 2 (S)-amino-6-boronohexanoic acid is shown as sticks with orange carbons. Below each loop has represented the alignment of Loop A and B from agmatinases and arginases.

A comparison of available crystal structures for arginases (ARGs) from human, rat liver, *Bacillus caldovelox*, and agmatinase (AGM) from *Deinococcus radiodurans* reveals an essential difference in the extension of two loops that contribute to forming the active site and containing residues that interact with the alpha carboxyl group of arginine in arginases (Figure 1). These two loops, named as loops A and B, are located at the entrance of the active site, and for the long loop (loop A), we replaced the residues present in EcAGM (Thr154 to Asp162) with those corresponding to the human arginase loop (Ile129-Leu140). This loop A included Asn130, a key residue for arginine specificity in hARG and in the structural equivalent position, a Tyr155 residue is found in EcAGM (Figure 1). In Table 1, mutants 1 to 3 show different chimeras between hARG and EcAGM, which also include a three residues insertion (LTT), present in hARG, to increase the size of the loop in EcAGM. The mutant T154I/Y155N/A156T/N157P-insLTT of EcAGM was not able to hydrolyze arginine and maintained its ability to hydrolyze agmatine, but with a *k*_cat_ that decreased slightly two times and K_M_ that increased about ten times. This has as consequence a decrease in *k*_cat_/K_M_ of almost two orders of magnitude. Furthermore, when all loop A’s residues were replaced in EcAGM (G158T/C159S/E160G and F161N/D162L), the same parameters were obtained, in comparison with the other mutants, without detectable activity with arginine.

Mutations of loop B residues (^188^TEFD^191^) in the EcAGM (mutants 4 and 5 in Table 1) generated a significant decrease in the activity, given mainly by a decrease over 100 times in the *k*_cat_ and ten times increase of the K_M_, which shows that loop B in EcAGM is related to catalysis, in addition to substrate binding. None of the combination of chimeras from both loops was sufficient to observe activity with arginine (mutants 6 and 7 in Table 1), while also a worse activity for agmatine was observed, but these mutants for both loops show some compensation in the loss of *k*_cat_ and K_M_ respect to the loop B mutants alone. 

Despite the changes made into loops A and B and both, none of the mutants could hydrolyze arginine, which would indicate that the structural information analyzed is not extrapolated to EcAGM (Figure 1). Therefore, to analyze residues that participate in the active site of the EcAGM and to explain the high specificity of the enzyme for agmatine, we performed crystallization assays to obtain the structure of the EcAGM.

### 2.2. Structural Analysis of E.coli Agmatinase: Monomer Architecture and Oligomerization State

To obtain the structure of EcAGM, we carried out crystallization tests in the presence of guanidine (Gnd) chloride 100 mM, obtaining crystals in two different conditions. The crystals showed adequate diffraction pattern allowing to solve two structures at resolutions of 1.8 Å (PDB entry 7LOL) and 3.2 Å (PDB entry 7LOX) (Table 2). For 1.8 Å resolution structure, the asymmetric unit of the crystal contains one chain of protein, an urea molecule, and agmatine in the channel formed by the loops A and B, and three manganese atoms; two at the active site, and one at the interfaces with the other two asymmetric units (Supplementary Figure S1). While for 3.2 Å structure (PDB entry 7LOX), the asymmetric unit contains three chains, two Mn2+ ions per chain, and guanidine at the active site.

**Figure 2:**
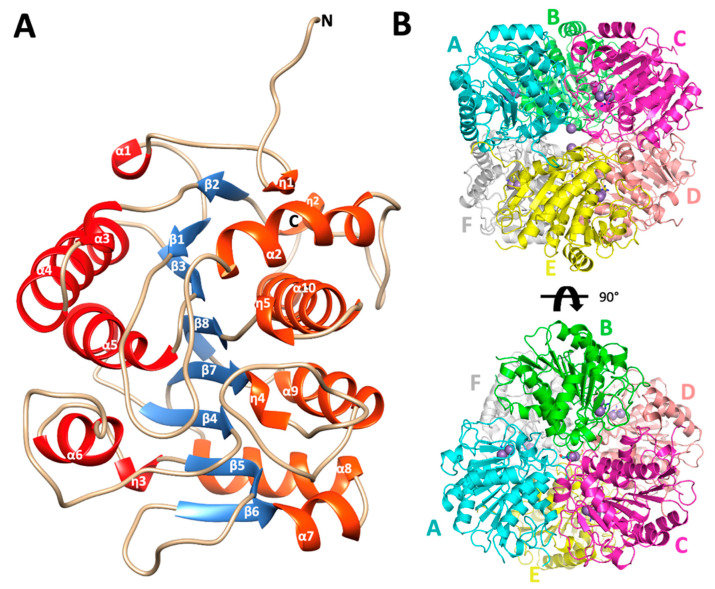
Monomer architecture and quaternary structure of *Escherichia coli* agmatinase. (**A**) The overall structure of the monomer of EcAGM. The eight central β strands are represented in blue. In red and orange have been represented both sides of helices that connect the β sheet. (**B**) quaternary structure of EcAGM, each monomer is labeled with letters from A to F. The arrangement of a dimer of trimers (ABC and DEF) in the hexamer is shown.

The overall fold of the monomers of EcAGM shows a single α/β/α sandwich domain, a central parallel eight β-sheet flanked on both sides by ten alpha-(α) and five 310-(η) helices, as is illustrated in Figure 2A. The strands of the β-sheet are arranged in the order β2-β1-β3-β8-β7-β4-β5-β6. Helices connect one strand to the next in such a way that a group of six helices (α1, α3, α4, α5, α6, and η3, in red, Figure 2A) resides on one side of the β-sheet and another group of nine helices (η1, η2, α2, α7, α8, η4, α9, η5, α10, in orange, Figure 2A) resides on the other side. According to a search using DALI, the structural superposition with the whole PDB shows similarity to other ureohydrolases, having the highest structural similarity with the putative agmatinase from *Burkholderia thailandensis* (BtAGM) (PDB entry 4DZ4). A characteristic insertion of structural motif for agmatinase between the α2 and the η2 helices is also present in the EcAGM structure, forming a β-turn between the carbonyl group of Arg72 and the amino group of Trp75, but with a different three-dimensional conformation found in agmatinase of *D. radiodurans* (DrAGM) [21] Commonly, two cis-peptide bonds have been described to maintain the structure and configuration of the active site of ureohydrolases, as it has been reported for DrAGM [21]; the first is presented between Pro64-Pro65 at the beginning of the β-turn α2-η2 and the second between Gly118-Gly119, as part of the conserved motif GGDH, which is reported as necessary for Mn^2+^ binding in ureohydrolase superfamily [30]. Interestingly, in EcAGM structure, the residues Pro64 and Pro65 are not conserved, presenting the Trp68–Glu69 dyad in structural equivalent position instead, but in the same β-turn α2-η2, a cis-peptide is found between Phe73-Pro74, showing that the cis peptide in this region would be a ubiquitous feature in agmatinases enzymes. In EcAGM, this structural motif (α2-η2) is implicated in the interaction with the neighboring subunit at the region of the active site. The other cis-peptide is conserved in EcAGM, presents in the Gly-Gly dyad of ^123^GGDH^126^ motif.

**Figure 3:**
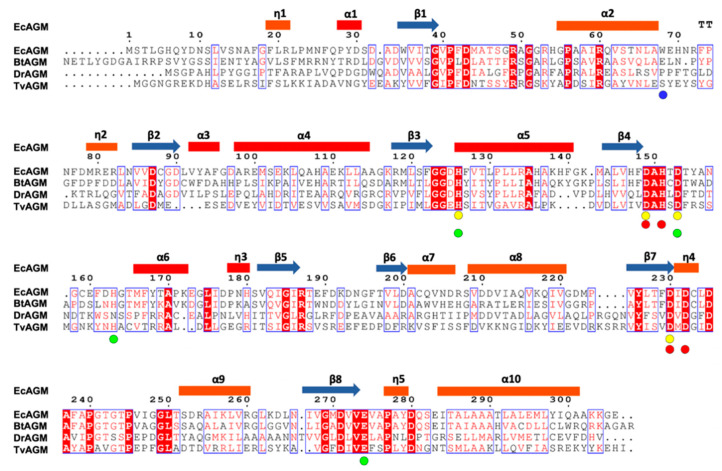
Sequence alignment of agmatinases from EcAGM, Burkholderia thailandensis (BtAGM), Deinococcus radiodurans (DrAGM), and Thermoplasma volcanium (TvAGM). With circles are indicated the amino acids implicated in the interaction with the Mna (yellow) and Mnb (red), respectively. In green circles are indicated the amino acids implicated in urea and guanidine binding. In blue is indicated the Trp68 in EcAGM.

PISA analysis of EcAGM structures indicates that the hexamer is the most stable oligomeric state for both structures 7LOL and 7LOX (Figure 2B and Supplementary Figure S2), presenting as monomer and trimer in the asymmetric unit, respectively (rmsdCα = 0.47 Å for monomers of 7LOL and 7LOX). The hexamer as oligomeric state tallies as is described to other bacterial agmatinases and arginases from bacteria and plants [21,31,32]. For the structure 7LOL (Figure 2B), the hexamer has a total buried surface of 23,560 Å2, and a ∆G_diss_ of 58.8 Kcal/mol, consistent with the oligomeric association observed in DrAGM and other prokaryotic ureohydrolases [28,32]. The contributing interactions to the hexamer conformation can be divided into those stabilizing a trimer (ABC or DEF) and the inter-trimers that conform to the hexamer (ABC-DEF). Two types of intra-trimer interactions can be observed, one formed by the association between two adjacent monomers (monomer-monomer interaction, i.e., A-B) and the other where one Mn^2+^ ion and TRIS molecule are found, implicating the interactions of the three monomers (trimeric interactions). Between two adjacent subunits, Trp68, Glu69, His70, Asn71, Arg72, and Phe73 from α2-η2 loop of one subunit interact with His151 from the loop β4-α6 and Arg187, Thr188, and Glu189 from the adjacent one (Supplementary Figure S3A). This interface participates in the active site entrance, and an agmatine molecule is found interacting with Trp68 from the neighboring subunit (Supplementary Figure S3B) in the structure 7LOL. However, this agmatine is in a non-catalytic position, away from the site of the Mn^2+^ ions, where an urea molecule is present. The presence of this agmatine probably comes from the expression and its location does not allow visualize a clear role in catalysis, possibly being an artifact of crystallization. Other interactions are given by residues Gln281, Ser282, and Glu283 of loop η4-α10 with Arg49 and Gln291 and the backbone of Tyr279 of the same loop from adjacent subunits (Supplementary Figure S3C). Residues from the helix α-10, Ser282, Leu287, Ala288, and Thr291 interact with the hydrophobic patch, formed by Pro236, Pro240, Pro245, and Ile247 from the loop η5-α9 of the neighboring subunit (Supplementary Figure S3D). These prolines form a conserved motif among analyzed agmatinases in the sequence alignment (Figure 3).

Regarding the trimer association, a 3-fold symmetry axis is formed with a hydrophobic interface formed by Ala237-Phe238 (η4-α10 loop) of each subunit. H-bonds mediate a similar intermonomer association between the backbone from Pro240 and Ala237 and the OH- of a TRIS buffer molecule. The η4-α10 loop is given by the strict conservation of the motif ^230^DX_2_DX_3_PX_4_P^245^, which is relevant for the oligomerization in the superfamily, essential for monomer-monomer and trimeric association. Interestingly, in the structure 7LOL, an interaction of Gln281 from three monomers in the trimers (ABC and DEF) coordinates an ion, attributable to a Mn^2+^ ion according to the anomalous density map (Supplementary Figure S1). However, this anomalous density is not observed in the structure 7LOX, nor the electron density of the 2FoFc map that allows assigning any ion. In other bacterial agmatinases, the Gln281 is semi-conserved (there are Gln and Asn), but in DrAGM, a Pro residue is found in a structural equivalent position. In BtAGM, this Gln281 residue is conserved, but in its structure (PDB 4DZ4), a water molecule was modeled in the position of the Mn^2+^ ión found in EcAGM 7LOL. Future studies could provide information on the relevance of this Mn^2+^ ion in the oligomerization of bacterial agmatinases.

Regarding the association between trimers to form the hexamer, the N-terminal ends of two chains (modeled residues 11 to 26) of different trimers are projected to the other, forming a structure of the interchange segments type (Supplementary Figure S4), which has already been reported in DrAGM [21]. Another relevant interaction between trimers is formed by helix α-2 with the same helix of the adjacent subunit forming a network of hydrogen bonds between His54 and Gln61 between both subunits (Supplementary Figure S4). Another interaction is given by a double symmetry between two loops β1-α2, governed mainly by Arg53, whose side chain contributes with H-donors towards the main chain of Thr46, Ser47, and Gly48 (Supplementary Figure S4). It is important to note that it is common to find a positive charge trait (Arg/Lys) in agmatinase in this position, which is not conserved in arginases, where Glu or Asp are found [21].

### 2.3. Guanidine, Urea, and Mn^2+^ Binding-Sites: Implications in the Catalytic Mechanism

**Figure 4:**
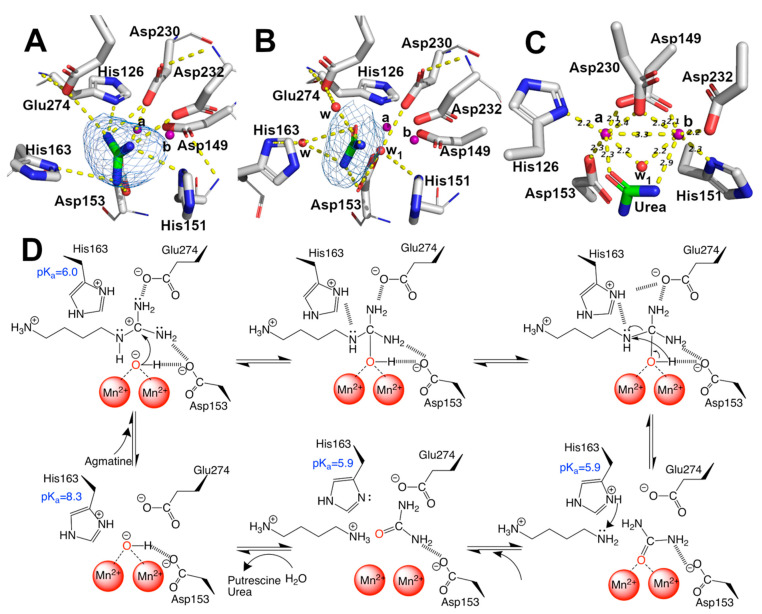
Mn^2+^, guanidine, urea binding-site, and catalytic mechanism. (**A**–**C**) guanidine, urea, and Mn^2+^ binding sites, respectively. Omit maps are shown as blue mesh contoured at 3.0 sigma for guanidine and urea. Polar interaction in (**A**,**B**) and distances in (**C**) were calculated with Pymol 2.0. Sidechains are shown as sticks, and the carbon atom of protein in white, the main chain, is shown as a line. Ligand is shown as stick, and carbon is colored in green. Mn^2+^ ions are shown as purple spheres and water as red spheres, indicating Mn_a_ and Mn_b_. (**D**) proposal of catalytic mechanism of EcAGM, polar contacts are shown as dashed lines and pK_a_ showed were estimated with PropKa3.1.

EcAGM contains one active site per monomer, and Gnd was found in 7LOX (Figure 4A), urea in 7LOL (Figure 4B), and both structures presented a cluster of two manganese at the active site (Figure 4C). The binuclear manganese cluster named Mn_a_ and Mn_b_ is located in the edge of the central β-sheet from the β7 strand and lidded by residues from the loop β1-α1, β4-α6, and η4-α9 (Figure 2A). Mn^+2^ ions are required and conserved in all ureohydrolases, and its binding site is highly conserved through the family of metallohydrolases (Figure 3) [20]. In both EcAGM structures (7LOL and 7LOX), Mn_a_ and Mn_b_ are separated by ~3 Å, and Mn_a_ is coordinated by His126, Asp149, Asp153, and Asp230. Mn_b_ instead is coordinated by Asp149, His151, Asp230 and Asp232. Mn^2+^-bridging water (w_1_) is possible to identify between Mn^2+^ ions in the high-resolution structure, which has been described as an essential nucleophile for ureohydrolases catalysis [20]. However, in 7LOX is not possible to identify the w_1_ due to the low resolution, but, likely, a water molecule is also found there as is found in other agmatinase’s structures (BtAGM PDB 4DZ4). In EcAGM, a high and a low-affinity site for Mn^2+^ ion have been described, and Salas et al. identified that His126 and His151 are bound to Mn^2+^ ion of the low and high-affinity site, respectively [24]. Therefore, after dialysis with 5 mM EDTA, Mn_a_ could be removed, while Mn_b_ not, which allows retaining almost half agmatinase activity after dialysis [18].

The solved structures in complex with guanidine and urea provide insights into the catalytic mechanism of the EcAGM (Figure 4D). The primary interaction of guanidine occurs with, His126, Asp153, His163, Asp230, Asp232 and Glu274 (Figure 4A), whereas urea can interact directly only with Asp153, Asp230, and Glu274 (Figure 4B). In prior studies, Asp153 has been proposed as a critical residue to form an H-bond with w1 and guanidine moiety, suggesting a critical role in the orientation and stabilization of the w1, allowing an optimal attack on the guanidinium carbon [26]. Also, Glu274 has been described as critical for catalysis and guanidine motif binding only, E274A mutant has only 1–2% of the activity of the wild-type enzyme and increase the K_M_ values for agmatine and K_i_ of guanidine, but do not alters the K_i_ for putrescine, indicating a role in the union and orientation of the guanidine group [19]. Finally, it has been shown that His163 plays a critical role in catalysis without participating in substrate-binding [25], where H163F mutant reduces the activity to 3–5% of wild-type, but it does not alter K_M_ for agmatine or K_i_ for putrescine. 

With the previous information reported for EcAGM and the structures reported here, we can propose a catalytic mechanism for EcAGM (Figure 4D): deprotonated Asp153 and Glu274 could establish polar interaction with guanidinium (Figure 4A), and due to the presence of Mn^2+^ ions significantly decrease the pK_a_ values of the groups around its, as shown by pKa estimates for 7LOL and 7LOX structures (Supplementary Table S1), the coordination of w1 with Mn^2+^ would allow decrease the pKa of water, resulting in a metal-bound hydroxide coordinated by both Mn^2+^/Asp153 and allowing to acts as a nucleophile on the carbon of the guanidinium. The last allows initiating a series of electronic rearrangement, where agmatine N4 atom can be stabilized by His163 and after hydrolysis the same His163 could act as a proton shuttle, in agreement with the previously proposed function of His163 as a stabilizer of the transition state and a proton donor to epsilon-amine group of putrescine before product dissociation, also promoting the ionization of manganese-bound water [25]. With 7LOX and 7LOL structures, which represent a substrate (guanidine) and a product conformation, it is possible to obtain information of His163 regarding its different conformations (Figure 4A,B) and estimate the pK_a_ in different states (Supplementary Table S1). This analysis allowed us to understand how this histidine is protonated in an APO state (pKa ~ 8) and then acts as an acid in the substrate-enzyme complex (pKa = ~6). Finally, putrescine and urea would leave the active site randomly, in agreement with the Uni–bi rapid equilibrium random kinetic mechanism reported for EcAGM [19]. Interestingly, His163 is conserved in other putative agmatinases (i.e., BtAGM and TvAGM), even is conserved in mammalian and plant arginases [31]. However, the only exception is the agmatinase from *D. radiodurans*, in whose position an asparagine is found, but since there are no kinetic characterizations of DrAGM, it is not possible to know the kinetic characteristics of this enzyme.

### 2.4. Analysis of Agmatine Binding: Molecular Dynamic Simulation and Role of Loop A and B of EcAGM

**Figure 5:**
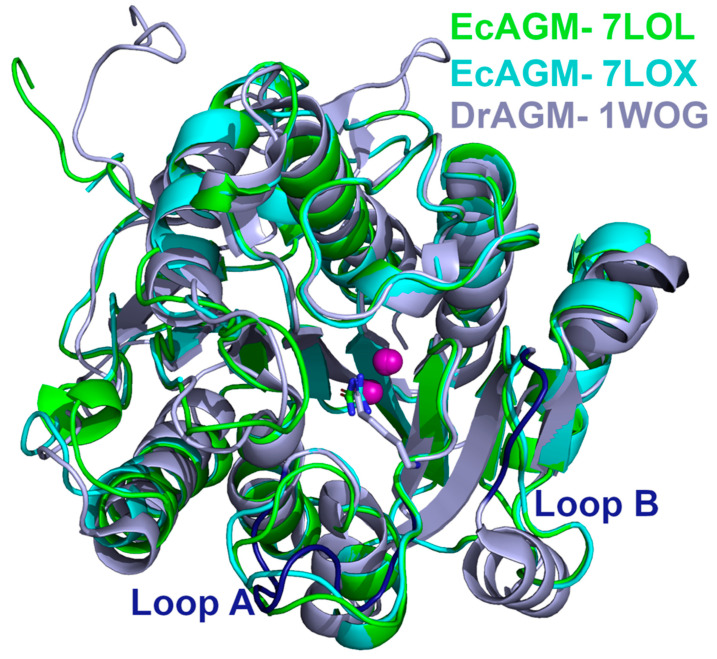
Structural comparison of EcAGM and DrAGM. EcAGM 7LOL and 7LOX are shown as green and cyan cartoons, respectively. DrAGM (PDB 1WOG) is shown as light blue, and its loops A and B are colored in dark blue. Guanidine, urea, and hexane-1,6-diamine are shown as sticks. Mn^2+^ ions are shown as purple spheres.

Considering the results of the A and B loop mutants in EcAGM (Table 1), based on the comparison between DrAGM and the human arginase (Figure 1), 7LOL and 7LOX structures were compared with the DrAGM (Figure 5). The structural superposition of EcAGM and DrAGM shows a clear difference in loops A and B. For loop A, the 7LOL and 7LOX structures of EcAGM have somewhat different conformations, while DrAGM has loop A in a highly distinct orientation. On the other hand, loop B of the 7LOL and 7LOX structures have the same conformation, but in DrAGM, loop B has a different orientation, which would indicate that the agmatine binding mode of EcAGM and DrAGM might be different.

**Figure 6:**
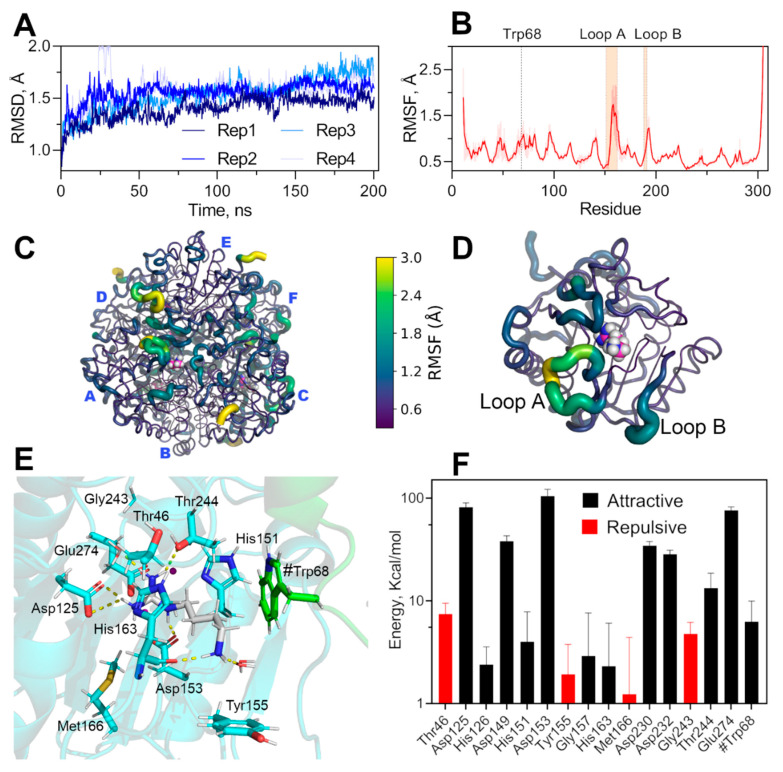
Molecular dynamic simulation of hexameric EcAGM and non-bonding interaction energy of agmatine binding-site. (**A**) RMSD of four replicates of 200 ns each. (**B**) RMSF for CA of EcAGM, mean and standard deviation calculated for six monomers and four replicates are shown. (**C**,**D**) RMSF mapped on hexameric and monomer structure, respectively. Structures are shown as cartoon putty according to the RMSF values scale (0.3–3 Å); the last five C-terminal residues are not shown. (**E**) Agmatine binging site after energy minimization, residues of the monomer analyzed are shown in cyan, agmatine is shown in white and green the neighboring monomer’s residues. Polar interactions are shown as yellow dashed lines and were calculated with Pymol 2.0. (**F**) Potential interaction energy calculated with MD simulations for residues at 5 Å of agmatine. Mean and standard deviation are shown. Only residues with a mean of over 1 kcal/mol are shown.

To evaluate agmatine binding mode in the active site of EcAGM, we have docked agmatine taking as reference the position of urea of the EcAGM structure and superimposing the structure of DrAGM (1WOG) with hexane-1,6-diamine, which reconstructs the position and orientation of the agmatine molecule in the active site of EcAGM. In the same way, six agmatine were docked on the EcAGM hexamer, and the system was solvated, neutralized with sodium ions, and energetically minimized using the *ff14SB* force field in Amber18 (Figure 6). The minimized complex shows that the amino group of agmatine only performs a polar interaction with the carbonyl of His163, in addition to some molecules of solvation water (Figure 6E). Other residues relatively close to the amino group are Thr46 and Ser47 of the β1-α1 loop, but they are located about 6 Å away. Interestingly, the agmatine’s amino group is located in a hydrophobic pocket formed by Tyr155 and Trp68, where Trp68 residue is part of the α2-η2 loop of the neighboring monomer—raising the hypothesis that the agmatine binding site comprises two monomers and that the aromatic residues Tyr155 and Trp68 would interact with the agmatine amino group through a cation-π system.

To evaluate the conformational dynamics of the hexamer’s active sites and the conformational stability of the pocket formed by Tyr155 and Trp68 in the presence of agmatine, we performed molecular dynamics calculations under NPT conditions. We have explored over 800 ns of total simulation through 4 replicates of 200 ns (Figure 6A). The simulations showed that the binding pocket is stable throughout the whole simulation and replicates, whereas the loops A and B showed high conformational dynamics, evidence by high values of alpha carbon RMSF (Figure 6B–D). The high conformational dynamics of loops A and B without permanent polar interactions of the amino group of agmatine during the simulations and the stability of the hydrophobic pocket indicates a particular agmatine binding mode. The dynamic of these loops would be relevant for the binding, and modification of this conformational landscape would alters the affinity for agmatine, explaining the high K_M_ for agmatine in the mutants of loops A and B (Table 1). Indeed, this agrees with kinetics parameters of loop A’s mutants, where K_M_ is increased over ten times with only a 50% decrease of *k_cat_*, the latter probably related to a change in the correct dynamics of the catalytic residue His163, residues located at the end of loop A.

The loop B’s mutants also increase the K_M_ for agmatine over ten times, which also could be related to the dynamic of these loops, but an overwhelming 100-fold decrease in *k_cat_*. A careful inspection of the determined structures and molecular dynamics trajectories evidence a permanent interaction between the loop B and Mn’s ligands ^149^DAHXD^153^ motif (Supplementary Figure S5), Thr188 of loop B interacts with the carbonyl of the Ala150 main chain, a neighboring residue to Asp149, His151, and Asp153. The Thr188 mutation could interfere with the correct orientation of Asp149/His151/Asp153, which would explain the decrease in *k_cat_* for these loop B mutants.

Calculations of the non-bonding interaction energy between agmatine and neighboring residues at 5 Å, show that Trp68 presents significant attractive energy, showing that the contribution of this residue belonging to the adjacent monomer would be relevant for the binding of agmatine and probably responsible for the high specificity for agmatine because arginine could not bind to this pocket with π-systems due to its carboxyl group. Tyr155 could also contribute to agmatine specificity, although the average potential energy found in the simulations is positive (repulsive), indicating some selective pressure to keep this aromatic residue in that position.

Analyzing the alignment of Figure 3, Trp68 is not conserved in other putative agmatinase reported structures. However, the α2-η2 loop conformation is similar in BtAGM and TvAGM (Supplementary Figure S6), and these structures present glutamic residues in a structurally equivalent position, which could stabilize the amino group of agmatine. In DrAGM, the α2-η2 loop has an entirely different orientation, which it hardly interacts with the agmatine binding site of the neighboring monomer. Future studies will be necessary to evaluate the role of the α2-η2 loop in the specificity/promiscuity of these bacterial agmatinases.

Interestingly, neighboring monomer residues’ contribution to the formation of agmatine binding pocket appears not to be fully conserved in agmatinases, neither is a widely reported characteristic of ureohydrolases. It has recently been reported that plant’s arginases (*Arabidopsis thaliana* and *Medicago truncatula*), residues of the equivalent α2-η2 loop of the neighboring subunit, participate in binding of the substrate arginine [31].

## 3. Conclusions

Structural information on agmatinases is scarce. A first approximation by comparing the structures from the human arginase and the bacterial agmatinase from *D. radiodurans* (DrAGM) allows to identify two loops in the active site entrance (A and B) as the main difference between both active sites of these enzymes. Chimeras with human arginase loops A and B inserted into *Escherichia coli* agmatinase result in mutants with a low affinity for agmatine (loop A mutant) or low catalysis (loop B mutant). However, none of them presented the capability to hydrolyze arginine. The determination of two structures of EcAGM in the presence of guanidine and urea allowed us to analyze the conserved architecture of the ureohydrolase and the interactions present between the monomers in the hexameric oligomerization state of this enzyme. Interactions among those formed by the α2-η2 loop with active sites residues of the neighboring monomers and analysis through MD simulations showed that Trp68 from the α2-η2 loop participates in the interaction with the agmatine amino group, probably stabilizing the substrate through a pi-cation system. MD simulations and analysis of loops A and B’s mutants showed that these loops’ conformational dynamics are essential for agmatine affinity. The structures with guanidine and urea made it possible to propose EcAGM’s catalytic mechanism and provide evidence of the importance of His163 as a proton shuttle and a stabilizer of the transition state in bacterial ureohydrolase.

## 4. Materials and Methods

### 4.1. EcAGM Expression and Purification

EcAGM’s gene, cloned into the histidine-tagged pQE60 vector, was expressed in *E. coli* strain BL21(DE3) [26]. Bacteria were grown at 37 °C in Luria Broth in the presence of ampicillin (100 µg/mL). The expression was induced during 4 h with 1 mM IPTG after reaching 0.5 AU at OD_600_. Cells were centrifugated at 8000× *g* for 10 min, cell resuspended in lysis buffer (50 mM Tris-HCl_,_ 500 mM NaCl, 1 mM PMSF), then sonicated and centrifuged at 35,000× *g* for 20 min. The soluble cell extract was loaded onto a 5 mL HisTrap HP column (GE Healthcare, Little Chalfont, UK), equilibrated with the re-suspension buffer: 50 mM Tris-HCl_,_ 500 mM NaCl, 40 mM Imidazole, pH 8.0. For elution, a continuous flow of the re-suspension buffer with a linear gradient of imidazole from 40 mM to 500 mM was used. The eluted enzyme was concentrated and re-purified by size exclusion chromatography S-200 column in a buffer, 25 mM Tris-HCl pH 8.0, 2 mM MnCl_2_.

### 4.2. EcAGM Site-Directed Mutagenesis

The mutants of EcAGM were obtained using the QuikChange^®^ Site-Directed Mutagenesis Kit (Stratagene) with the plasmid pQE60 containing the EcAGM cDNA as the template. Serial mutations were made in each of the loops. In summary, the nucleotides present in the EcAGM sequence were replaced by the corresponding nucleotides of the human ARG type l sequence. The LTT insertion and the F190 deletion were introduced by the same strategy using the previously mutated AGM gene as template DNA. The presence of the desired mutation and the absence of unwanted changes were confirmed by automated DNA sequence analysis.

### 4.3. EcAGM Activity Determination and Kinetics Experiments

Routinely, EcAGM activities were determined by measuring the formation of urea (product) using 80 mM agmatine in 50 mM glycine–NaOH (pH 9.5) and 5 mM MnCl_2_. All the assays were initiated by adding the enzyme (final concentration of 5–10 nM), to the substrate, buffer, and MnCl_2_ solution previously equilibrated at 37 °C, and urea was determined by forming a colored complex with α-isonitrosopropiophenone [29], measuring the absorbance at 540 nm. Initial velocity studies were performed in duplicate and repeated three times using increasing agmatine concentrations between 0.2–50 mM. Kinetic parameters were obtained by fitting the experimental data to the appropriate Michaelis–Menten equation using nonlinear regression with Graph Pad Prism version 7.0 for Windows (Graph Pad Software Inc., San Diego, CA, USA). Protein concentration was determined using the standard Bio-Rad protein assay (Bio-Rad, Irvine, CA, USA) with bovine serum albumin as standard.

### 4.4. X-ray Crystallography

After size-exclusion chromatography the protein was concentrated to 12 mg/mL in Buffer 25 mM Tris-HCl pH 8.0, 2 mM MnCl_2_, 100 mM guanidine chloride. Sitting drop vapor diffusion experiments were performed by mixing 0.8 µL protein solution and 0.8 µL of crystallization condition and equilibrating against over 70 µL mother solution at 18 °C. After one week, we observed crystals formation in two conditions: i. 0.1 M phosphate/citrate pH 4.2 and 40% PEG 300 and ii. 0.2 M Lithium sulfate monohydrate, 0.1 M HEPES pH 7.5, 25% *w/v* Polyethylene glycol 3350. Crystals in condition (i) were cooled by plunging them into liquid nitrogen without cryoprotection. However, in condition (ii), we used glycerol 15% with the same mother solution as cryoprotectant. Diffraction data were collected at the beamline 23-ID-B at the Advanced Photon Source, APS (Chicago, IL). Data were indexed and integrated with XDS [33] and scaled and merged with Aimless [34]. Phasing was performed by molecular replacement with BALBES [35] using the structure PDB ID 3NPI. The model was manually refined using Coot [36] and Phenix Refine [37] from the suite PHENIX [38]. Different ligands (urea, guanidine and agmatine) were incorporated using coot, and the refinement process was used as an indication of rightness. Interestingly, in 7LOL agmatine was found in the entrance of active site form by loop A and B, which probably come from *E. coli* expression. In 7LOL, discrimination between guanidine and urea was made based on refinement (R_free_, Real-space correlation in Phenix) and difference map. Initially, a guanidinium molecule was modeled in the active site, but the difference map showed a positive value of difference-map in one of the guanidinium nitrogen atoms. Considering the possible hydrolysis of guanidinium to urea at pH 4.2 [39], we refined with urea instead of guanidinium showing the best results in the difference map and refinement parameters. The stereochemistry of the structures was validated with MolProbity [40].

### 4.5. Sequence and Structural Analysis

Protein sequences of agmatinases from *Escherichia coli*, *Burkholderia thailandensis, Deinococcus radiodurans*, and *Thermoplasma volcanium* were aligned with clustalW using the matrix Blosum62. Structural visualization was performed with UCSF Chimera [41], and Pymol Molecular graphics Systems, Version 2.0 (Schrödinger, LLC), and the analysis of oligomerization states was performed using PISA [42]. Estimation of pK_a_ was calculated with PROPKA3.1 [43] using default parameters.

### 4.6. Molecular Dynamic Simulation of EcAGM in Complex with Agmatine

EcAGM hexamer of 7LOL crystal structure determined by PISA was used as initial coordinates to build the molecular systems. Superposition of EcAGM monomer with urea and DrAGM (1WOG) in the presence of hexane-1,6-diamine allowed to reconstruct the agmatine molecule in Pymol 2.0 (Schrödinger LLC, New York, NY, USA). The protonation states of ionizable residues were assigned using the Propka3.1 software [44]. Parameters derived from the *ff14SB* force field were used for protein [45], parameters for manganese ions were taken from [46] available in Amber parameters database, and parameters for guanidine and 2-Amino-2-hydroxymethyl-propane-1,3-diol were estimated with Antechamber [47] using GAFF force field [48]. The hexameric complex was solvated in a TIP3P water orthorhombic box [49] with a 12 Å extension over the protein surface using *tleap* from Ambertools19 [50]. Counter-ions (Na^+^) were used to maintain electroneutrality. Energy minimizations were carried out following four successive stages of minimization, wherein each minimization cycle consisted of 5000 steps using the steepest descent algorithm followed by 5000 steps using a conjugated gradient method. In the first two-stage, a harmonic positional restraint of 500 kcal/mol*Å^2^ was applied first over the all-protein atoms to accommodate the solvent and ions, then were applied only to heavy protein atoms to allow to minimize the hydrogens. In the following two stages, minimizations were carried out successively, reducing the restraint from 10 to 0 kcal/mol*Å^2^, except for a restriction of 50 kcal/mol*Å^2^ between Mn^2+^ ions and the carbon atom of the guanidine group of agmatine. The minimized systems were equilibrated under NVT conditions, heating the system from 0 K to 298.15 K using the weak-coupling Berendsen thermostat [51,52] in a window of 250 ps. Then, 20 ns-long equilibrations in NPT conditions were carried out for each system, keeping a constant temperature of 298.15 K using the Berendsen thermostat and constant pressure of 1 atm using the Berendsen barostat. Finally, four independent replicates with productions of 200 ns were performed for each system under the same conditions of the NPT equilibration with the restraint only in the Mn^2+^ ions and the carbon atom of guanidine to allow exploration of the conformations of the primary amine region of agmatine. All the simulations were performed using pmemd.cuda of the Amber18 software [50], using periodic boundary conditions with a time step of 2 fs. The SHAKE algorithm [53] for bond length constraints involving hydrogen atoms was used. Non-bonded interactions were calculated using a cut-off of 8 Å, and the Particle Mesh Ewald method [54] was used for treating long-range electrostatic interactions. RMSD, RMSF, and non-bonding potential energy interactions were calculated using *rms*, *rmsf*, and *lie* functions, respectively, available in *cpptraj* [55].

## Figures and Tables

**Table 1:** Kinetic constants of *E. coli* agmatinase mutants of loop A and B.

Mutants	Arginine			Agmatine		
	*k_ca_*_t_ (s^−1^)	K_M_ (mM)	*k_cat_*/K_M_ (M^−1^ s^−1^)	*k_cat_* (s^−1^)	K_M_ (mM)	*k_cat_*/K_M_ (M^−1^ s^−1^)
Wild type	ND			120	1.1	1.1 × 10^5^
Loop A mutants						
(1) T154I-Y155N-A156T-N157P-/ins L-T-T	ND			55	12	4.6 × 10^3^
(2) T154I-Y155N-A156T-N157P-/ins L-T-T/-G158T-C159S-E160G	ND			48	15	3.2 × 10^3^
(3) T154I-Y155N-A156T-N157P-/ins L-T-T/-G158T-C159S-E160G-F161N-D162L	ND			46	18	2.5 × 10^3^
Loop B mutants						
(4) T188D-E189V	ND			0.96	12	8 × 10^2^
(5) T188D-E189V-/ΔF190	ND			0.24	6.5	3.7 × 10^1^
Mutants in both loops						
(6) T154I-Y155N-A156T-N157P-/ins L-T-T/-G158T-C159S-E160G-F161N-D162L-T188D-E189V	ND			7.3	7	1 × 10^3^
(7) T154I-Y155N-A156T-N157P-/ins L-T-T/-G158T-C159S-E160G-F161N-D162L-T188D-E189V-/ΔF190	ND			8.4	8	1.1 × 10^3^

L-T-T: Insertion of the amino acids leucine, threonine, and threonine to enlarge the loop. The values of the kinetic parameters indicated in this table correspond to the results of two experiments performed in duplicate, and the standard deviations were not greater than 5%. ND, not detected activity.

**Table 2:** Data collection and refinement statistics of crystal structures.

	EcAGM-Urea (7LOL)	EcAGM-Gnd (7LOX)
Wavelength	1.033	1.771
Resolution range	35.79–1.8 (1.86–1.8)	64.51–3.2 (3.31–3.2)
Space group	R 3 2:H	P 31 2 1
Unit cell	81.746 81.746 207.436 90 90 120	129.028 129.028 88.29 90 90 120
Total reflections	499876 (47706)	564172 (55308)
Unique reflections	25154 (2475)	14314 (1418)
Multiplicity	19.9 (19.3)	39.4 (39.0)
Completeness (%)	99.87 (99.88)	99.87 (100.00)
Mean I/sigma(I)	28.76 (3.61)	26.70 (4.25)
Wilson B-factor	27.1	84.6
R-merge	0.06576 (0.892)	0.1561 (1.066)
R-meas	0.06753 (0.9161)	0.1582 (1.08)
R-pim	0.01521 (0.2075)	0.02515 (0.1718)
CC1/2	1 (0.933)	0.999 (0.929)
CC*	1 (0.983)	1 (0.982)
R-work	0.1702 (0.2355)	0.1727 (0.2256)
R-free	0.1947 (0.2617)	0.2172 (0.2679)
Number of non-hydrogen atoms	2432	6539
macromolecules	2259	6517
ligands	24	22
solvent	149	0
Protein residues	294	852
RMS (bonds)	0.015	0.012
RMS (angles)	1.35	1.40
Ramachandran favored (%)	99.32	98.33
Ramachandran allowed (%)	0.68	1.67
Ramachandran outliers (%)	0.00	0.00
Rotamer outliers (%)	1.28	0.00
Clashscore	3.11	9.98
Average B-factor	33.35	81.50
macromolecules	32.90	81.51
ligands	34.70	76.68
solvent	39.81	-

Statistics for the highest-resolution shell are shown in parentheses.

## Data Availability

The structures of EcAGM have been deposited in the Protein Data Bank (PDB) with the following codes: 7LOL and 7LOX.

## Supplementary Materials

The following are available online at https://www.mdpi.com/article/10.3390/ijms22094769/s1.
