# Supplementary material for: Crystal Structure of Escherichia coli Agmatinase: Catalytic Mechanism and Residues Relevant for Substrate Specificity
Source: Int J Mol Sci. 2021 Apr 30;22(9):4769. doi: 10.3390/ijms22094769 (PMC8125230; doi:10.3390/ijms22094769)
Supplement: Supplementary file 1 [file ijms-22-04769-s001.zip › ijms-1154542-supplementary.pdf]

Supplementary Materials

Crystal structure of *Escherichia coli* agmatinase: catalytic mechanism and residues relevant for substrate specificity.

Pablo Maturana[1], María S. Orellana[2], Sixto M. Herrera[1], Ignacio Martínez[3], Maximiliano Figueroa[3], José Martínez-Oyanedel[3], Victor Castro-Fernandez*[1], Elena Uribe*[3].

[1]Departamento de Biología, Facultad de Ciencias, Universidad de Chile, 7800003, Ñuñoa, Santiago, Chile.

[2]Facultad de Ciencias de la Vida, Universidad Andres Bello, 8370251, Santiago, Chile.

[3]Departamento de Bioquímica y Biología Molecular, Facultad de Ciencias Biológicas,

Universidad de Concepción, Casilla 160-C, Concepción, Chile.

*Corresponding authors:

Elena Uribe, 56-41-2204428, e-mail: auribe@udec.cl,
Victor Castro-Fernandez, 56-2-29787332, e-mail: vcasfe@uchile.cl

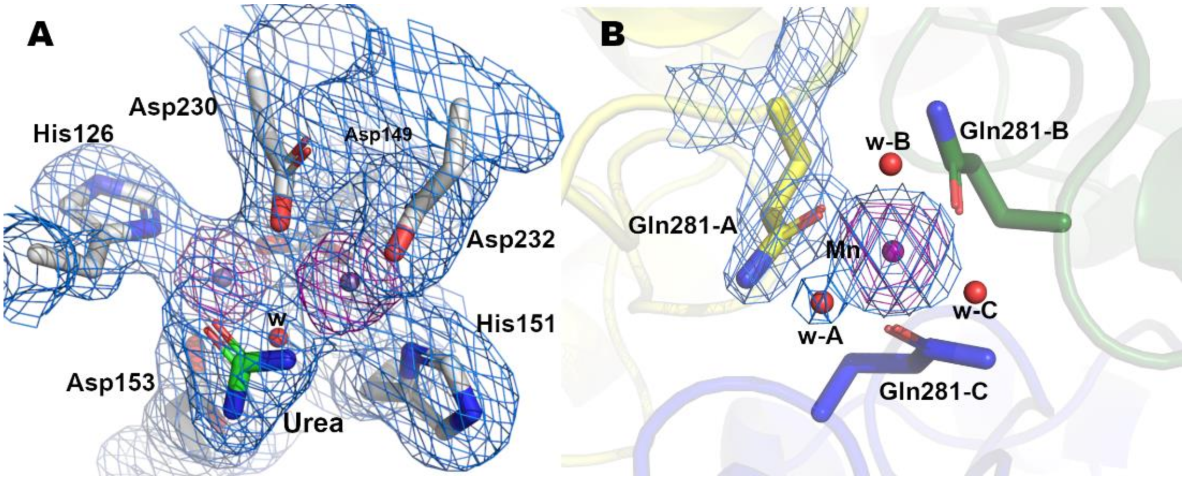

Supplementary Figure S1. Anomalous map and position of Mn^{2+} ions. (A) Mn2+ binding sites at the active site. (B) Mn2+ binding site at the trimer interface. Mn2+ ions are shown as purple spheres. The anomalous map is shown as purple mesh contoured at 3.0σ. 2Fo-Fc map is shown as blue mesh contoured at 1.5σ. Water molecule is labeled as w.

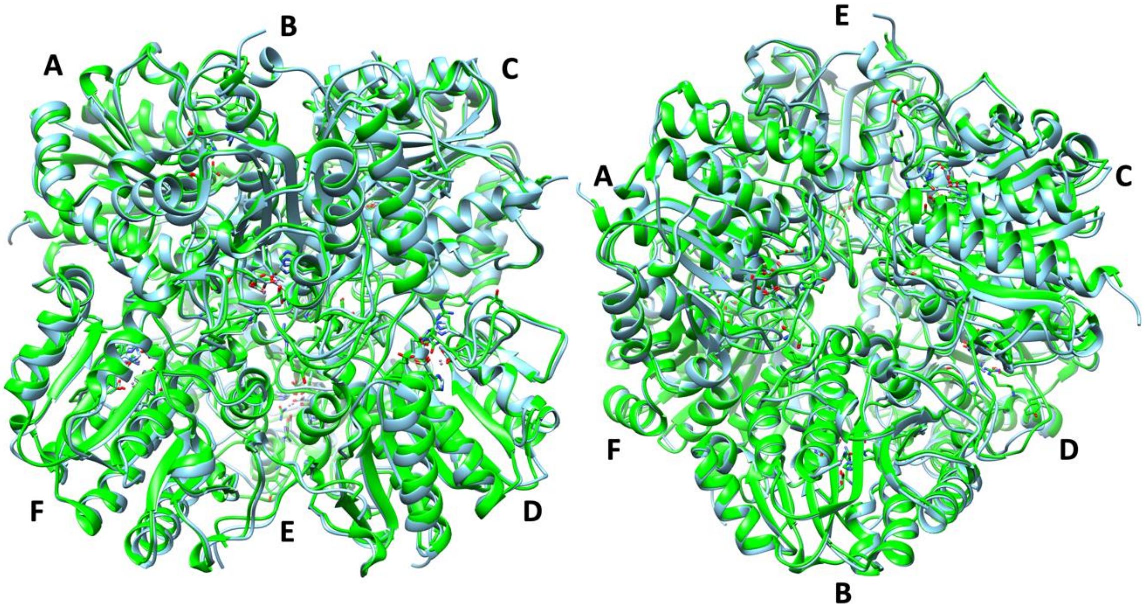

Supplementary Figure S2. Structural superposition of the oligomeric assembly of EcAGMs. We estimated the oligomeric assembly for Agmatinases 7LOL (light blue) and 7LOX (green) using PDBePISA v1.52. In the Left panel, the trimer ABC and DEF are shown up and down in the structure, respectively. In the right panel, the hexamer rotates 90° around the axis of the association between both trimers.

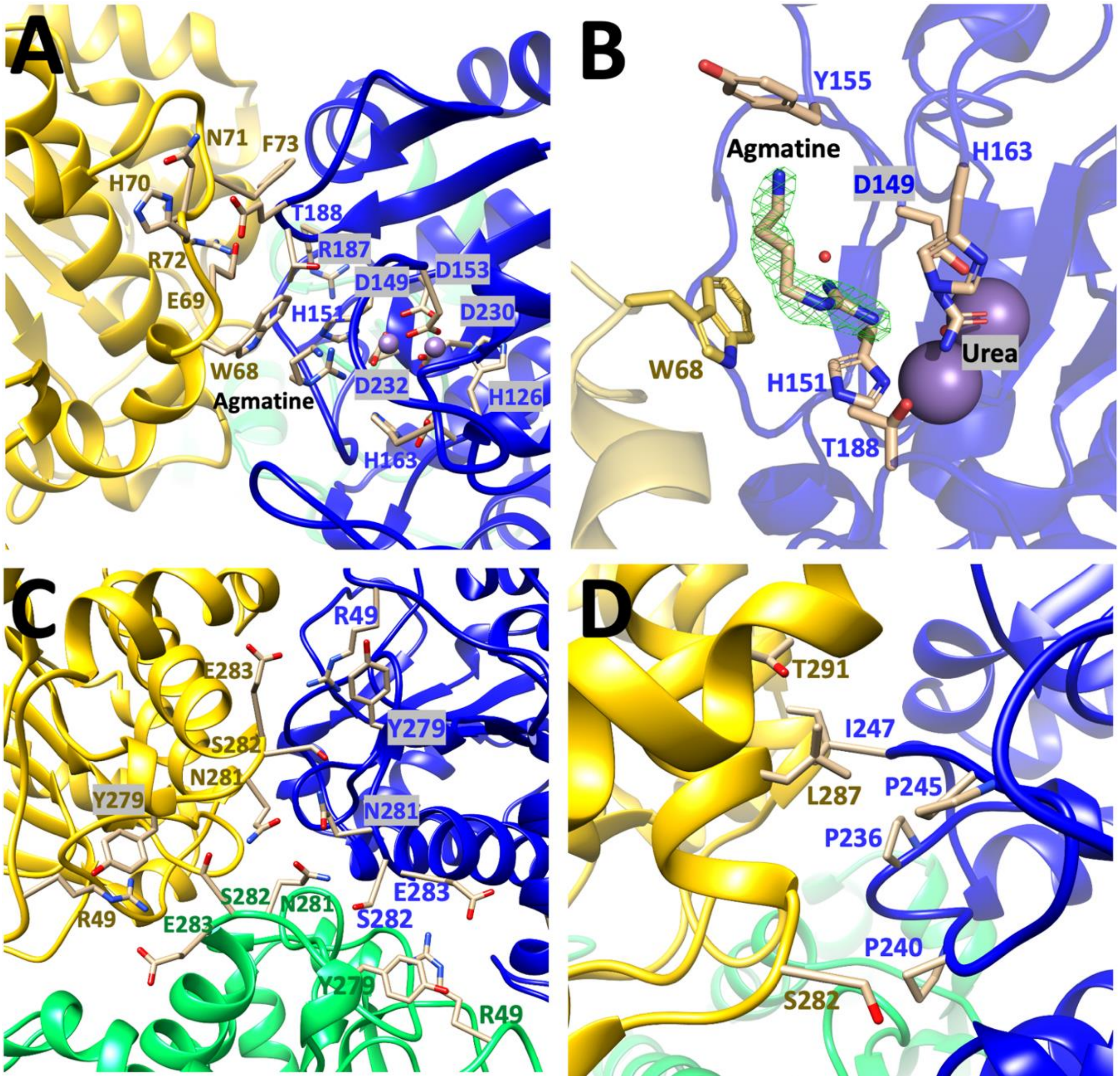

Supplementary Figure S3. Intra-trimer interface interactions. The three primary interfaces that occur in the association of a trimer are shown. A. shows the association between residues of the α2- η2 loop with the adjacent catalytic site entrance. (B) the interaction is given by Trp68 with agmatine (omit map shown as green mesh at 3σ) at the neighboring subunit's active site entrance. (C) Interaction between three subunits is shown by the association of residues from the loop η5- α10 from each chain. (D) Association interface between residues from the α10 and residues from the proline-rich loop η4-α9 from the adjacent subunit in the trimer.

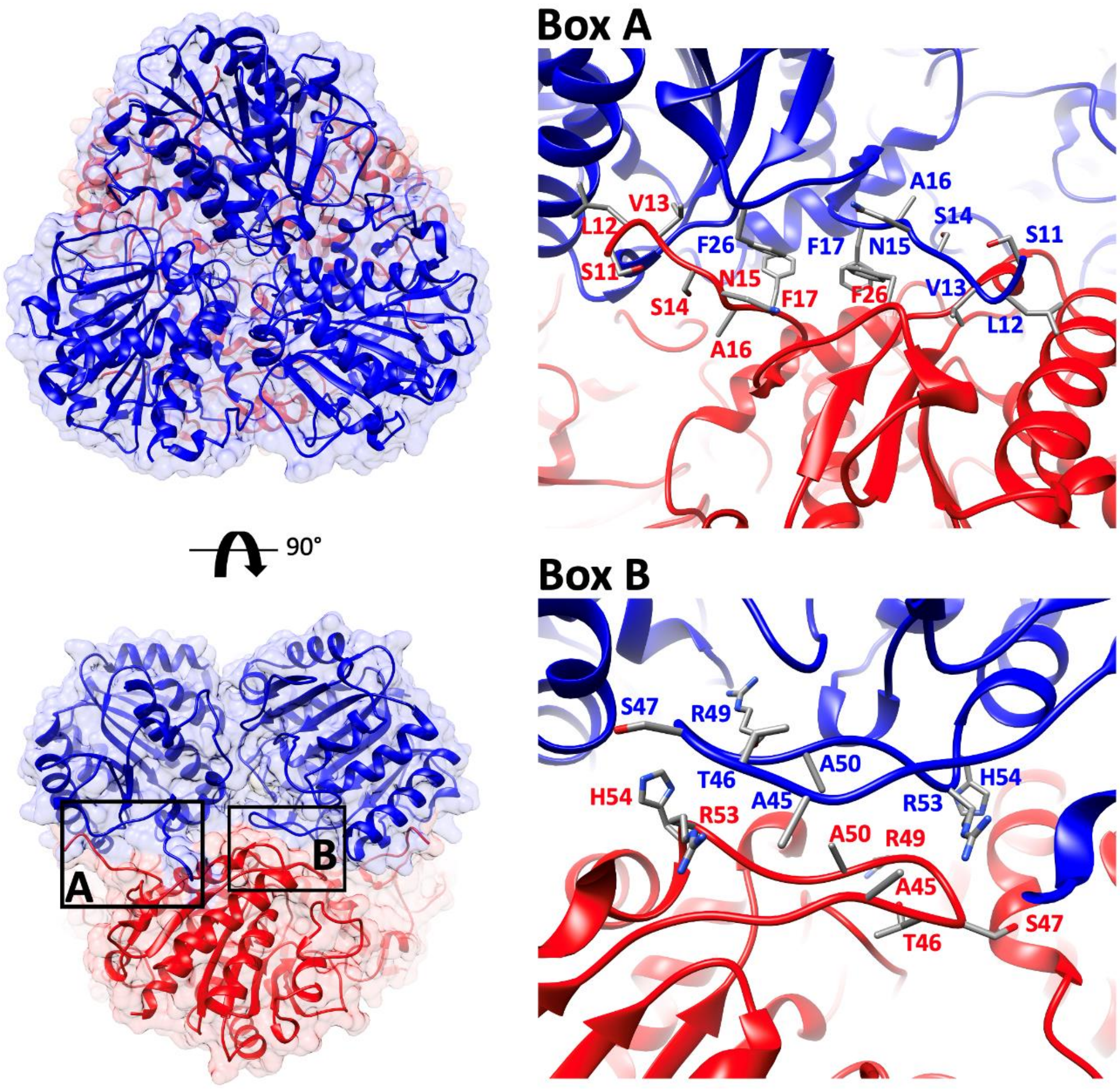

Supplementary Figure S4. Inter-trimer interface interactions. the hexamer colored by each trimer in red (ABC) and blue (DEF) in the left panel is shown. Below, the hexamer was rotated at 90 degrees. The main two inter-trimer associations are indicated in boxes A and B. In the box A, the association is given by the interaction of both N-terminal loops from adjacent subunit from each trimer. In the box B, the interface is given by the interaction of both β1-α1 loops from adjacent subunits from each trimer.

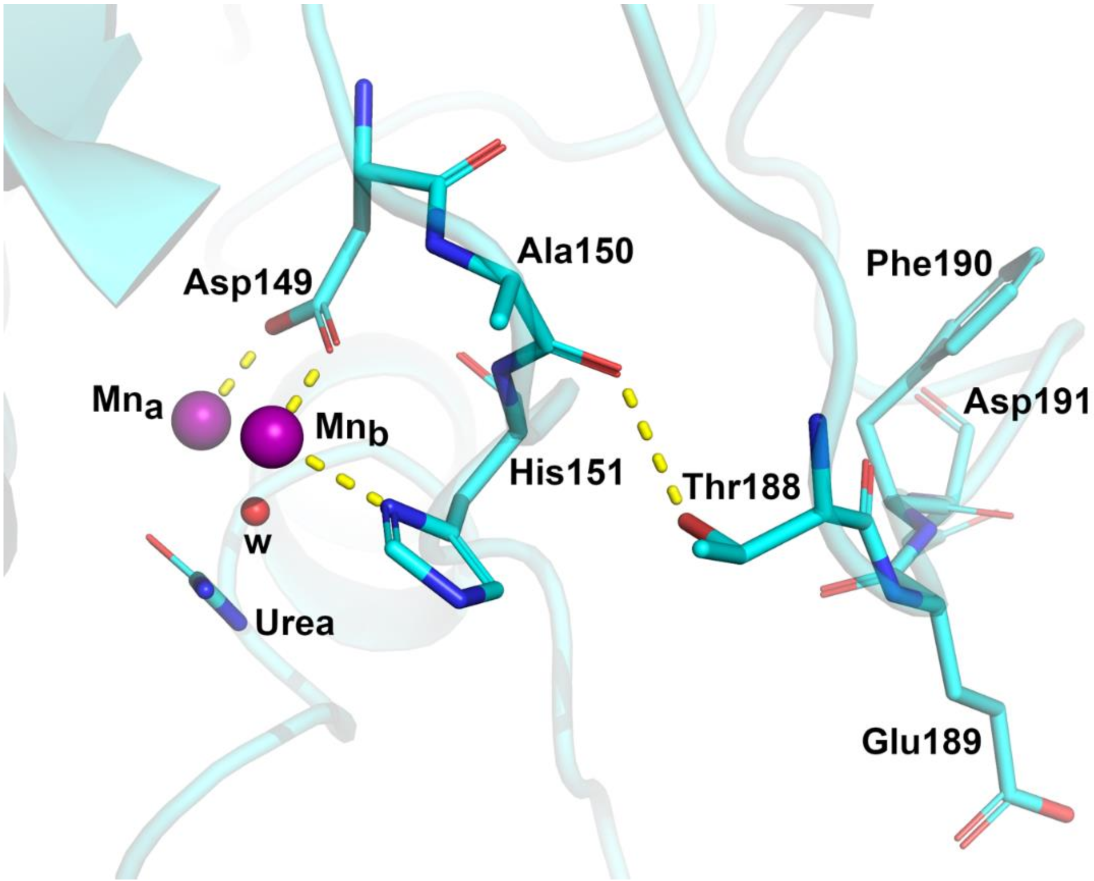

Supplementary Figure S5. Loop B interaction with manganese ligand motif ^{149}DAHXD^{153}. Thr188 interacts with the main chain carbonyl of Ala150 of ^{149}DAHXD^{153} motif, which is highly conserved in ureohydrolases and essential for manganese binding.

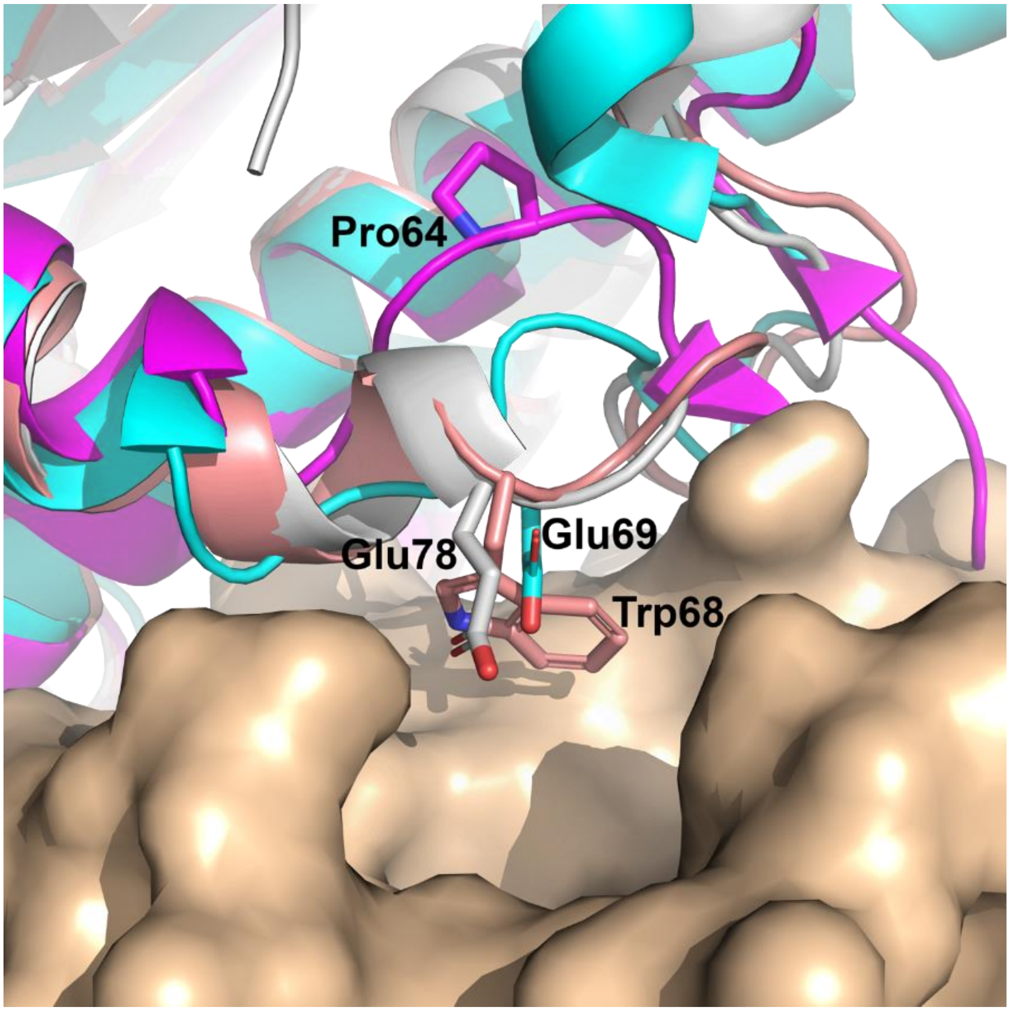

Supplementary Figure S6. Interaction between the entry of the active site and the α2-η2 loop of different bacterial agmatinases. α2-η2 loop from EcAGM (Ligh pink), BtAGM (White), TvAGM (Cyan) and DrAGM (Magenta). Residues in the structural equivalent position of Trp68 of EcAGM are shown as sticks.

Supplementary Table S1. Estimation of pK_a for active site residues with PROPKA3.1

	7LOL – Products conformation			7LOX* – Substrate conformation		
Residue	All	Wo AGM/ Urea	Wo Mn^{2+}/ligand	All	Wo Mn^{2+}/ligand	pKa of reference
Asp149	-12.8	-11.0	7.1	-8.0	0.3	3.8
Asp153	-5.9	-2.5	1.0	-2.0	4.4	3.8
Asp230	-3.8	-1.4	10.4	-0.4	12.1	3.8
Asp232	1.7	2.7	2.1	-0.3	4.0	3.8
Glu274	0.5	6.2	8.3	2.8	6.8	4.5
His126	8.0	8.6	14.3	8.4	14.6	6.5
His151	4.0	6.4	12.2	5.4	12.0	6.5
His163	5.9	8.3	10.1	6.0	8.0	6.5

*mean of three chains (ABC).

pKa values were estimated with whole structures (all), the structures without a specific ligand (i.e., Wo Gnd), and without any ligand (Wo Mn^{2+}/ligand).
